# Chitosan-Based Mucoadhesive Vaginal Tablets for Controlled Release of the Anti-HIV Drug Tenofovir

**DOI:** 10.3390/pharmaceutics11010020

**Published:** 2019-01-05

**Authors:** Raúl Cazorla-Luna, Fernando Notario-Pérez, Araceli Martín-Illana, Roberto Ruiz-Caro, Aitana Tamayo, Juan Rubio, María Dolores Veiga

**Affiliations:** 1 Departamento de Farmacia Galénica y Tecnología Alimentaria, Facultad de Farmacia, Universidad Complutense de Madrid, 28040 Madrid, Spain; racazorl@ucm.es (R.C.-L.); fnotar01@ucm.es (F.N.-P.); aracelimartin@ucm.es (A.M.-I.); rruizcar@ucm.es (R.R.-C.); 2 Instituto de Cerámica y Vidrio, Consejo Superior de Investigaciones Científicas, 28049 Madrid, Spain; aitanath@icv.csic.es (A.T.); jrubio@icv.csic.es (J.R.)

**Keywords:** vaginal preexposure prophylaxis of HIV, tenofovir controlled release, mucoadhesive vaginal tablets, natural polymers combination, chitosan, pectin, locust bean gum

## Abstract

Vaginal microbicides have the potential to give women at high risk of contracting HIV the option of self-protection by preventing the sexual transmission of the virus. In this paper, mucoadhesive vaginal tablets based on chitosan, alone and in combination with pectin and locust bean gum, were developed for the sustained release of tenofovir (an antiretroviral drug). The formulations were placed in simulant vaginal fluid (SVF) to swell, and Hg porosity and SEM microscopy were used for the microstructural characterization of the swelling witnesses. The results show that the association of pectin and chitosan generated polyelectrolyte complexes and produced a robust system able to maintain its structure during the swelling process, when small pores are formed. Drug release and bovine vaginal mucoadhesion studies were performed in SVF showing that tenofovir-controlled dissolution profiles and adhesion to the mucosa were conditioned by the swelling processes of the polymer/s in each formulation. Tablets based on chitosan/pectin have the most homogeneous tenofovir dissolution profiles and last up to 96 h, remaining attached to the vaginal mucosa for the same period. These formulations can therefore be considered a good option for the self-protection of women from the sexual transmission of HIV.

## 1. Introduction

According to the Joint United Nations Programme on HIV and AIDS (UNAIDS) factsheet from July 2018, between 31.1 and 43.9 million people currently live with HIV, although fewer than 23 million of them have access to antiretroviral therapy. 1.8 million people contracted the infection in 2017 indicating that, although the transmission of the disease has fallen by 47% since 1996, the problem is still far from being solved. The situation of young women is particularly worrying, as 7000 women between the ages of 15 and 24 become infected with the virus each week. In Sub-Saharan Africa particularly, this group is twice as likely to live with HIV as men, and 75% of new infections occur in women aged 15–19 [1].

This high incidence of HIV in young women can be attributed to age-disparate sexual relationships, poor negotiating power with respect to the use of preventive tools (such as condoms) and intimate partner violence; moreover, the lack of prevention systems for women does not allow them to protect themselves from HIV transmission. The development of effective systems to prevent HIV infection initiated by women themselves is a challenge that has yet to be solved, and pre-exposure prophylaxis (PrEP) is one of the most innovative tools for increasing the options for self-protection in the population at high risk of HIV infection [2,3].

Among the drugs used in vaginal microbicide development, reverse transcriptase inhibitors act on viral enzyme reverse transcriptase, which is responsible for transforming the viral RNA into DNA, thus inhibiting viral replication. Of these, tenofovir (TFV) has been shown to be effective and safe, and has a long half-life [4]. TFV gel for vaginal application proved effective in the CAPRISA 004 trial when used pericoitally, reducing women’s risk of infection by 39% compared to a placebo [5,6]. It has also been demonstrated that vaginal administration of TFV can achieve concentrations in vaginal tissue that are 130 times higher with an oral administration regimen [7].

Nevertheless, currently available vaginal dosage forms have several limitations, such as leakage, messiness and low residence time. As these issues lead to low adherence rates—the key factor in achieving protection [8]—novel dosage forms are required. For example, vaginal films for the release of TFV have been developed and have even reached clinical trials, the FAME-04. Films users reported less product leakage than gels users. However, moderate vaginal leakage was still observed and daily administration was required, pointing to the need for further work. More sophisticated polymer-based systems for the controlled release of TFV are currently in the early stages of development; these include freeze-dried bigels [9], hydrogels containing polymeric nanocapsules [10], spray-dried microspheres [11] or complex polymeric tablets [12]. All these systems propose the use of natural mucoadhesive polymers, which are a promising option for the development of biocompatible controlled-release drug-delivery systems [13].

Chitosan is a natural copolymer obtained by partial deacetylation of chitin, which is found in the exoskeleton of crustaceans, and also produced extracellularly by fungi and brown algae [14]. This polymer is composed of β1→4)-linked 2-acetamido-2-deoxy-β-d-glucopyranose (*N*-acetylglucosamine) and α(1→4)-linked 2-amino-2-deoxy-β-d-glucospyranose (glucosamine). The percentage of glucosamine determines its physicochemical properties [15], of which the most notable is its cationic nature, and especially interesting if we consider that most natural polymers present neutral or anionic charges [16]. The mechanical properties of chitosan-based systems can therefore be modified by forming a polyelectrolyte complex with anionic compounds [17]. This polymer offers several advantages from a pharmaceutical point of view, as it is biocompatible and biodegradable [18]. It has been used in the development of numerous systems for vaginal administration such as tablets, films and gels [19,20,21]. Formulations containing this excipient show sustained drug release and mucoadhesive properties [15,19,22,23,24], and it may also be useful in pharmaceutical systems for the prevention of STDs due to its intrinsic antimicrobial activity [25,26,27,28] and immunostimulant capacity [14]. As the loss of the formulation is the main limitation to its therapeutic efficacy [29], the association with other substances may offer a good alternative. The polymers than have been studied for use in combination with chitosan include locust bean gum [30], pectin [31], hydroxypropylmethylcellulose [29] and tragacanth gum [32] among others.

The development and evaluation of a formulation based on mixtures of chitosan and locust bean gum [30] has been previously reported for buccal administration; it was observed that the second is capable of buffering the effect of acid pH on the drug release from chitosan-based formulations [33]. This polymer is a neutral galactomannan vegetable gum obtained from *Ceratonia Silqua* L. [34,35]. It consists of a linear chain of (1→4)-linked-d-mannopyranosyl units with (1→6)-linked side chains of -d-galactose, in a mannose/galactose ratio of 4:1. In the pharmaceutical industry, it has shown potential in the inhibition of gastrointestinal diseases and has also been used as a carrier agent for the controlled release of drugs, either alone or in combination with other polymers [35].

Chitosan and pectin are able to form complexes through electrostatic interactions between positively charged amino groups in chitosan and negatively charged carboxylate groups in pectin [36]. This combination has been explored with promising results for the development of different dosage forms [37]. Pectin is a natural heteropolysaccharide obtained from apple or citrus peel. This polymer contains at least 65% galacturonic acid units. It is formed of (1→4)-linked α-D-galactosyluronic acid residues and neutral sugars such as rhamnose, galactose, arabinose and others. The acid groups of the galacturonic units can be methoxlyated and amidated to different degrees [38,39]. It has been assessed as an excipient in several vaginal formulations such as gels, tablets and moisturizers [13,40,41].

With this background, the aim of the present work was to develop chitosan-based vaginal mucoadhesive tablets for the controlled release of tenofovir by combining chitosan with other complementary polymers to achieve four-day release and mucoadhesion periods with moderate swelling profiles. This would be comfortable for the patient and ensure therapeutic compliance.

## 2. Materials and Methods

### 2.1. Materials

Tenofovir (TFV, lot: FT104801401, MW: 287.21 g/mol) was provided by Carbosynth Limited (Berkshire, UK). Chitosan (lot: 0055790), was supplied by Guinama (Valencia, Spain). Locust bean gum (lot: 010 M0087) and pectin (lot: BCBK7271V) were supplied by Sigma Aldrich (Saint Louis, MO, USA). Magnesium stearate PRS-CODEX (MgS; lot: 85269 ALP) was acquired from Panreac (Barcelona, Spain). All other reagents used in this study were of analytical grade and used without further purification. Demineralized water was used in all cases.

### 2.2. Polymers Characterization

Intrinsic viscosity is one of the characteristic values of polymers, and at preset conditions, only depends on the molecular weight of molecules [42]. Diluted gels of increasing concentrations of the three polymers were prepared (from 0.0125 g/dL to 0.1 g/dL). Locust bean gum and pectin gels were prepared in water [43,44]. As it does not gel in water, an aqueous solution of acetic acid 0.3 mol/L and sodium acetate 0.2 mol/L was used to prepare chitosan gels [45]. Their absolute viscosities (*η*) were measured with a Brookfield viscosimeter (Viscoelite, Fungilab^®^, Barcelona, Spain). Solvent viscosity (*η*_0_) was also measured. From η and *η*_0_, the reduced viscosities (*η_sp_*) were determined by the following equation (Equation (1)):

(1) $$\eta_{sp}=\eta -\eta_{0}/\eta_{0}$$

The intrinsic viscosity [*η*] was obtained from *η_sp_* of the different polymers, according to the Huggins equation (Equation (2)) [44]:

(2) $$\eta_{sp}/c=\left[\eta \right]+Hc$$

where *c* is the concentration of the gel n g/dL and *H* is a constant which depends on the polymer under evaluation. The relationship between intrinsic viscosity and molecular mass has been previously described by the Mark–Houwink–Sakurada equation (Equation (3)) [44]:

(3) $$\left[\eta \right]=K{\left(M_{w}\right)}^{\alpha }$$

where *K* and *α* are constants that depend on the polymer’s nature, solvent, and measuring temperature; *M_w_* is the viscosity-average molecular mass. All samples were measured at room temperature (25 °C).

The molar fraction of *N*-acetylglucosamine units in the chain of chitosan–referred to as degree of deacetylation–was experimentally determined in triplicate through the titration method proposed by Czechowska-Biskup et al. [46]. 0.100 g of chitosan was dissolved in 10 mL of hydrochloric acid 0.1 M and the solution was titrated with NaOH 0.1 M. (Equation (4)):

(4) $$NH_{2}\left(\%\right)=\left(\left(C_{HCl}\times V_{HCl}-C_{NaOH}\times V_{NaOH}\right)\times M_{w}/g\right)\times 100$$

where *C_HCl_* and *C_NaOH_* (mol/L) are the concentrations of HCl and NaOH, respectively; *V_HCl_* and *V_NaOH_ (L)* is the volume of HCL or NaOH employed, respectively; *M_w_* is the molecular weight (NH_2_ = 16 g/mol) and *g* is the weight of CH in g. Assuming that 9.94% is the theoretical NH_2_ percentage, the degree of deacetylation was calculated as follows (Equation (5)):

(5) $$DD\;\left(\%\right)=NH_{2}\left(\%\right)/9.94\times 100$$

As regards pectin, the ratio of esterified galacturonic acid groups to galacturonic acids groups—defined as the degree of esterification (DE)—was experimentally determined in triplicate attending to the method described by Liew et al. [47]. 0.200 g of pectin was dissolved in 20 mL hydrochloric acid 0.1 M and the solution was titrated with NaOH 0.1 M up to the complete neutralization of free acid groups, the result being the initial titration volume (ITV(mL)). Then, 10 mL of NaOH 0.1 M were added to neutralize polygalacturonic acid and allowed to stand at room temperature for 2 h to de-esterify pectin. 10 mL of HCl 0.1 M were added to neutralize NaOH and the sample was titrated with NaOH 0.1 M, the volume used being recorded as final titration volume (FTV (mL)). The DE was then calculated using the formula (Equation (6)):

(6) DE (%)=[FTV/(ITV+FTV)]×100

### 2.3. Preparation of the Tablets

Physical mixtures, whose composition is indicated in Table 1, were compacted at a pressure of 3.68 × 10^8^ Pa during 240 s, with a press similar to the one used to prepare of samples for the IR technique, and 13 mm dies. Blank batches (without drug) and loaded batches (with 30 mg and 100 mg of TFV) were obtained.

### 2.4. Tablets Characterization

Tablets were weighted and thickness, diameter and hardness of all the batches were measured in triplicate using a TA.XT*plus* Texture Analyser (Stable Micro Systems, Surrey, UK). Results were processed, and average values and standard deviations were calculated.

### 2.5. Swelling Test

The swelling behavior of the tablets was assessed using the method previously described by Ruiz-Caro et al. [48]. Each sample was fixed to a stainless-steel disc using a cyanoacrylate adhesive. This preparation was then placed in a beaker containing 100 mL of simulated vaginal fluid (SVF, pH = 4.2) [49]. The beakers were placed in a shaking water bath at 37 °C and 15 opm. To adequately characterize the swelling of the tablet, samples were weighed at preset times until the complete dissolution or erosion of the tablet. Each analysis was done in triplicate, and swelling ratio (SR) was determined according to the formula (Equation (7)):

(7) $$SR\left(\%\right)=\left\{\left[\left(W_{t}-W_{0}\right)/W_{0}\right]/P_{np}\right\}\times 100$$

where *W_0_* and *W_t_* correspond to the weight of the dry and swollen tablet respectively; and *P_np_* is the proportion of natural polymer, which is the swellable component of the tablet.

Maximum swelling ratios (*SR*_max_) and time to maximum swelling (*t*_max_) for each batch were then statistically processed through a two-way ANOVA considering polymer nature and drug content as factors (α = 0.05).

### 2.6. Characterization of Swelling Witnesses. Hg Porosimetry and SEM

Swelling witnesses were prepared for further structural characterization [19]. Tablets from each batch were fixed to stainless-steel discs and placed in beakers containing SVF. The preparation was maintained in the shaking water bath (37 °C, 15 opm) until the maximum SR was achieved for each formulation (data previously determined in the swelling test). The swollen tablets were then extracted and lyophilized (Lio-Labor^®^; Telstar, Barcelona, Spain), obtaining porous structures called swelling witnesses. The lyophilization conditions were identical for all the formulations, in order to guarantee that the differences in the porosities of the formulations are a consequence of their composition and not of the lyophilization process. In this way, the comparison among the different systems is justified.

The pore size distributions (PSD) of the witnesses were determined by mercury porosimetry using an Autopore II 9215 (Micromeritics Corp., Norcross, GA, USA). The corresponding PSD_m_ were calculated from the intrusion curves assuming cylindrical pore shapes in all cases. The swelling witnesses were observed by electron microscopy using a field emission scanning electron microscope (JEOL JSM-6335F, Tokyo, Japan).

### 2.7. Drug Release

The release study was performed according to the methodology proposed by Sánchez-Sánchez et al. [18]. Each tablet was introduced in a borosilicate glass flask containing 80 mL of SVF in sink conditions [19] and placed in a shaking water bath at 37 °C and 15 opm. At given times, 5 mL were removed from the flask and immediately replaced with 5 mL of tempered SVF. The aliquot was filtered and the concentration of TFV was determined by UV spectroscopy at a wavelength of 260 nm (Shimadzu^®^ UV-1700 spectrophotometer, Kyoto, Japan). The study was performed in triplicate and statistically processed by determining the similarity factor f2 [50].

### 2.8. Mucoadhesion Assessment

In order to determine the tablets’ capacity to adhere to the vaginal mucosa at the time of administration, the work and force necessary for detachment was assessed using the TA.XT*plus* Texture Analyser (Stable Micro Systems) through a new ex vivo method. The samples were fixed to a 20 mm stainless-steel probe. Square fragments of 2 × 2 cm of bovine vaginal mucosa (obtained from a local slaughterhouse) were fixed to a petri dish with a cyanoacrylate adhesive. The probe with the tablet was moved at a speed of 1 mm/s until it came into contact with the mucosa, applying a contact force of 500 g for 30 s. The probe was then separated from the mucosa at a speed of 0.1 mm/s until the complete detachment of the tablet. The force applied during the detachment of the formulation was measured at a rate of 500 pps. The force applied vs. the distance travelled by the probe was measured, and the maximum force required to separate the tablet from the mucosa was recorded. Also, the area under the curve between the force–distance profiles was determined [51]. Each batch was evaluated in duplicate, and the results were statistically processed using a two-way ANOVA, with drug dose and polymer as the factors (α = 0.05).

However, the tablets must remain adhered to the mucosa while the drug is being released, in order to afford the woman protection against the sexual transmission of HIV for the longest possible time. The optimal formulation must not only release the drug in a sustained manner, but also remain adhered to the vaginal mucosa during a similar period, so the residence time of the tablet in the vaginal mucosa was assessed through an ex vivo mucoadhesion test [19]. A sample of bovine vaginal mucosa was fixed with a cyanoacrylate adhesive to an 8.5 cm × 5 cm stainless steel plate. Each tablet was then adhered to the mucosa, applying a given pressure (500 g for 30 s). The preparation was placed at an angle of 60° inside a beaker containing SVF, and then in the shaking water bath (Selecta^®^ UNITRONIC320 OR, Barcelona, Spain) at 37 °C and 15 opm. The residence time was assessed by observation of the samples. All batches were evaluated in duplicate.

## 3. Results and Discussion

### 3.1. Polymers Characterization

**Figure 1:**
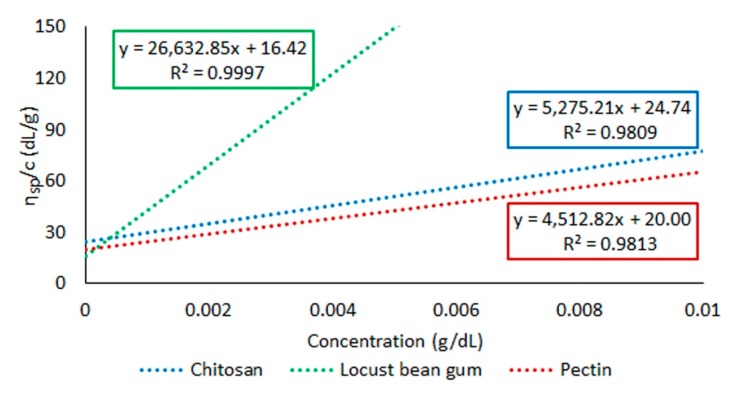
Plots of reduced viscosity, *η_sp_/c* (dL/g) vs. concentration (g/dL) for polymeric gels of chitosan, locust bean gum and pectin.

In Figure 1, *η_sp_*/*c* (dL/g) vs. concentration (g/dL) for the three polymers is displayed. All polymers showed a direct relation between *η_sp_*/*c* and the concentration of the gel, locust bean gum-based gels being the ones showing higher differences in viscosity depending on the concentration. Pectin- and chitosan-based gels show similar profiles, with a less steep slope compared to locust bean gum gels. However, it must be noted that chitosan does not gel in water, which makes these results hardly extrapolable to the performance of the tablet. The value of [*η*] was therefore obtained for all the polymers employing these plots. The *K* and α values for Mark–Houwink–Sakurada equation in each polymer had been previously described in literature, making it possible to determine *M*_w_, displayed in Table 2. DD (%) for chitosan and DE (%) for pectin are also shown in Table 2.

The molecular masses obtained for pectin and locust bean gum are within the normal range of molecular masses for both polymers [43,52]. These results indicate that the chitosan employed for the study was a low-molecular-weight chitosan [53] with a degree of deacetylation of (50.47–58.99)%. In the case of pectin, it was shown that it was a highly methoxylated pectin [54].

### 3.2. Tablets Characterization

Thickness, diameter and weight values for the batches are displayed in Table 3.

The thickness of the tablets was directly related to their weight. In all the cases, the thickness of the tablets was higher than 1.5 mm and lower than 2.5 mm. An average diameter of 13 mm was obtained in all cases due to the fact that the same dye was used in all cases. The average weights obtained fit with theoretical values for all the batches.

**Figure 2:**
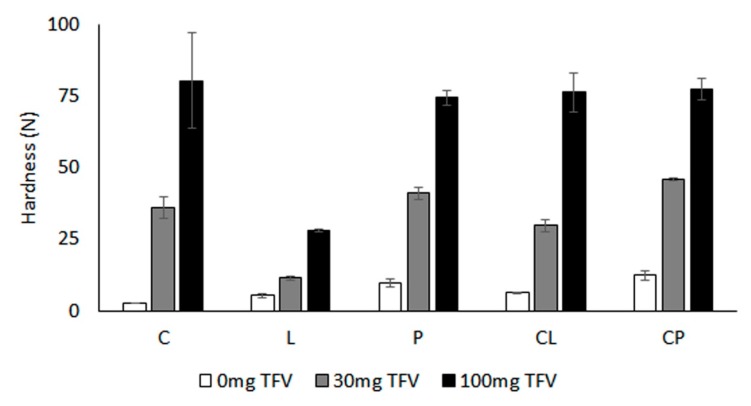
Hardness (N) obtained for the tablets.

Hardness data for the tablets evaluated are represented in Figure 2. Tablets constituted exclusively by polymers showed low hardness, lower than 10 N in all cases, due to the amorphous nature of these substances, which are not suitable for tableting without compaction excipients. However, the addition of a drug leads to a significant increase in hardness in all cases. The more TFV there is in the system, the higher the hardness of the tablet. This indicates that TFV, which is a crystalline substance, may act as structural agent, allowing the correct transmission of force from the punch to the mass being compacted. However, the degree of influence of the drug is significantly lower in the case of locust bean gum-based tablets, which may be due to a low interaction between the drug and the polymer. According to data, addition of TFV to the tablets significantly improves their handling, all loaded systems being suitable for handling and self-administration.

### 3.3. Swelling Test

**Figure 3:**
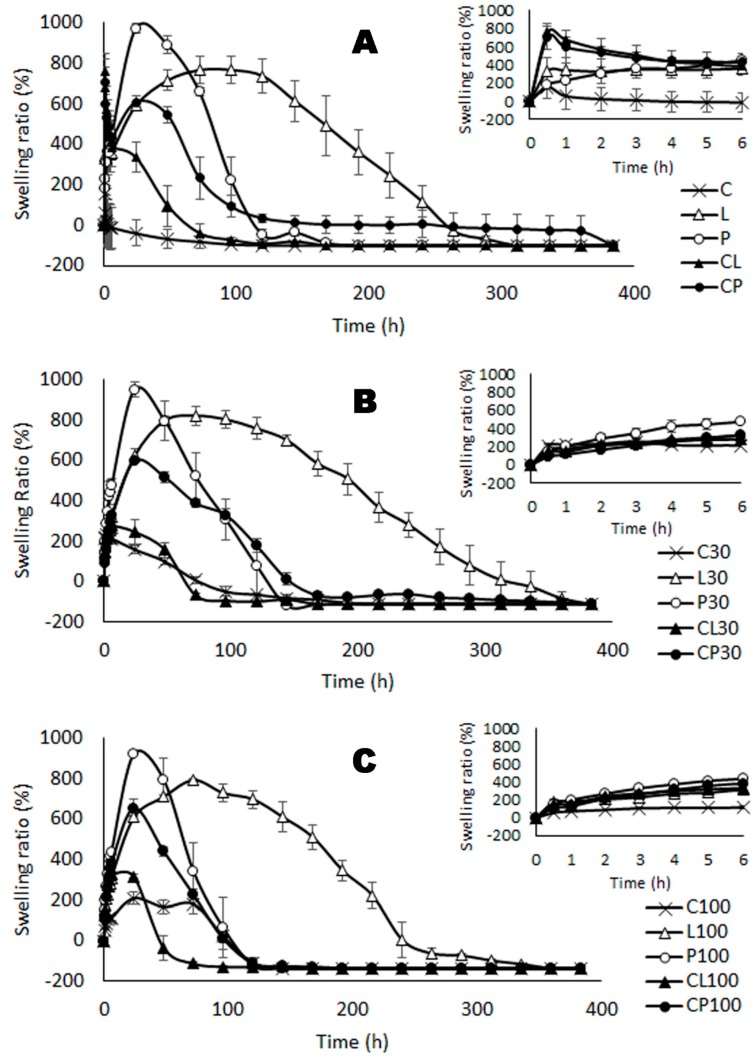
Swelling ratio values vs. time in the tablets containing no drug (**A**), 30 mg (**B**) or 100 mg (**C**) of TFV. The first 6 h are shown in detail in the small graphics in the top right corner.

Figure 3 shows the swelling profiles, where weight increases were observed in all cases due to the uptake of SVF in the tablets. After reaching the maximum SVF uptake (maximum SR, *SR*_max_), the weight of the formulations decreases due to the erosion or dissolution of the system in the medium. Nevertheless, the swelling profiles are conditioned by the nature of the polymer.

Figure 3A contains the swelling profiles of the blank batches. C tablets show a sudden entry of SVF in the first hours. However, the gelation of the polymer is incomplete in this medium, as gelling in chitosan is pH-dependent and require diluted acids [55,56]. The erosion of the tablet is observed after reaching a low maximum SR value after no more than 24 h. P tablets swell completely in this medium, adequately hydrating the tablet and forming a gel in 24 h. P tablets have the highest swelling rate, as pectin is an acidic polymer that is able to gel in this medium, whose pH is above the pKa of pectin (2.9–3.2) [31], which enables it to capture a large amount of SVF. The behavior observed in L batch is similar to that of the P batch, although in this case a more consistent gel is obtained [57], leading to more moderate swelling that is also more sustained over time. The swelling behavior in batches of tablets containing a combination of two polymers is conditioned by the nature of each polymer and the possible interaction between them. Thus, CL tablets show moderate swelling profiles with a lower dissolution time in the medium than observed when the polymers are evaluated separately. Locust bean gum is a neutral polymer that does not modify its charge in the medium, so the partial gelation of the chitosan induces the separation of the polymer chains in the locust bean gum, drastically reducing the high consistency of the gel. In contrast, CP tablets reveal a synergy between chitosan and pectin due to their ability to form polyelectrolyte complexes, as previously described [37]. Although the penetration of SVF is slower than in the tablets containing only pectin—and therefore has a lower maximum SR—more time is required for the complete dissolution of the system. Pectin releases protons into the medium, which remains negatively charged. Chitosan captures the protons released by the pectin and the chains of both polymers become joined by means of electrostatic interactions, generating a very compact structure which slows the entry of SVF.

Figure 3B shows the swelling profiles of the batches containing 30 mg of TFV. It can be seen that the entire swelling and erosion process is delayed for batch C30, indicating that the tablets containing TFV maintained their structure in SVF for a longer period, as the TFV acted as a structural agent that prevents the destructuring of the system. L30 and P30 tablets show overlapping swelling profiles for L and P respectively, because the presence of TFV is unable to alter the water uptake capacity of both polymers. This indicates that locust bean gum and pectin generate systems that are robust enough not to modify the physicochemical properties of the tablets in the presence of the drug. However, after mixing locust bean gum or pectin with chitosan, CL30 and CP30 show a delay in the swelling process that can be attributed to the structural behaviour of TFV in the presence of chitosan.

This behaviour is even more evident in Figure 3C, which shows the swelling data obtained from systems containing 100 mg TFV. C100 requires more time to imbibe SVF and erode in the medium, so the more TFV the C tablets contain, the greater the structuring action of the drug. The swelling profiles of L100 and P100 overlap with the corresponding systems containing no drug or 30 mg of TFV, so the drug is unable to modify the characteristic swelling behaviour of either of the two polymers. CL100 and CP100 batches show the structuring capacity of TFV on chitosan and their swelling and complete erosion processes are even more delayed compared to the systems containing either no drug or 30 mg of TFV.

**Figure 4:**
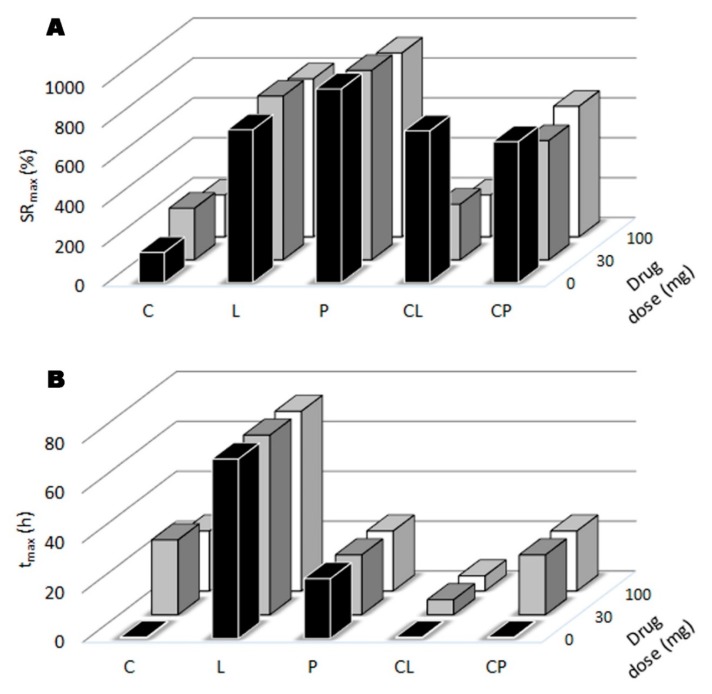
Maximum swelling ratio, SR_max_ (**A**) and time required to achieve *SR*_max_, *t*_max_ (**B**) for the tablets assayed.

Figure 4 contains the values of *SR*_max_ and *t*_max_ from the corresponding swelling profiles. No significant differences are obtained in the *SR*_max_ from the statistical analysis of C, C30 and C100 batches, but the presence of TFV delays the entry of SVF, so the greater the amount of drug in the tablet, the longer the *t*_max_, as the structuring power of the drug slows the entry of the medium in the chitosan tablet. No significant differences were detected in the statistical analysis between L, L30 and L100 in either the *SR*_max_ or in the *t*_max_, and the same true for the P, P30 and P100 batches. This suggests that the L and P formulations are robust and SVF can access the tablet as the characteristic three-dimensional arrangement of the polymer chains does not depend on the presence of TFV. When chitosan is combined with locust bean gum (CL, CL30 and CL100), the presence of the drug significantly reduces the *SR*_max_ and increases *t*_max_. This can be explained by the fact that the drug is interposed between the ungelled chitosan and the locust bean gum gel, so the entry of SVF is less abrupt and the separation of the locust bean gum chains is less marked; the amount of SVF that is able to enter the system is therefore lower and impeded, leading to a higher *t*_max_. In batches where chitosan is mixed with pectin (CP, CP30 and CP100), both polymers are joined by electrostatic interactions so *SR*_max_ is not modified by the presence of TFV, although *t*_max_ is delayed, as SVF entry is slowed by the structuring power of TFV.

### 3.4. Microstructure of Witnesses. Hg Porosimetry and SEM

**Figure 5:**
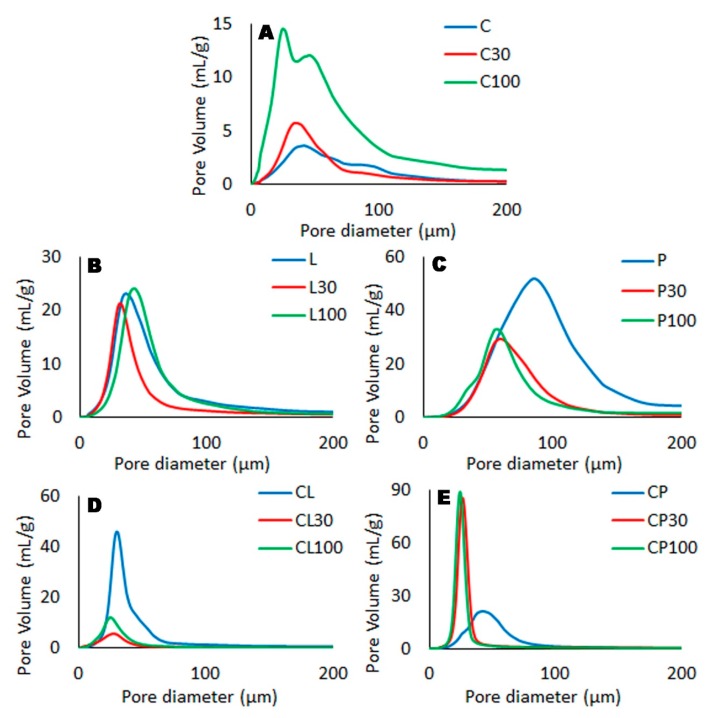
Results obtained from Hg porosimetry performed on the swelling witnesses of all the batches based on chitosan (**A**), locust bean gum (**B**), pectin (**C**), chitosan/locust bean gum (**D**) and chitosan/pectin (**E**).

**Figure 6:**
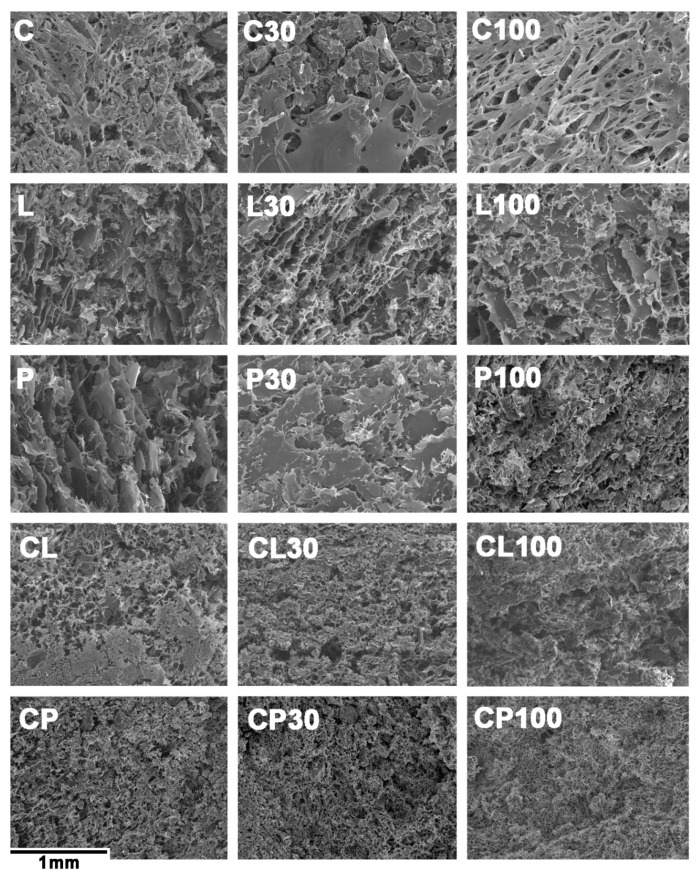
Images obtained through scanning electron microscopy (SEM) of the witnesses at 100 times magnification.

The PSD of the swelling witnesses samples are plotted in Figure 5, and the corresponding microstructures are shown in Figure 6.

All PSD are plotted in terms of their maximum pore volume (mL/g), and it can be seen that for the un-mixed polymers (chitosan, locust bean gum and pectin), the pore volume increases in the order C < L < P, agreeing with the data from the swelling test. However, when chitosan is mixed with locust bean gum or pectin the pore volume is in the order CL > CP, also due to the aforementioned swelling behaviour, as the PSD reflects the water present in the hollows at *t*_max_, and which was removed during lyophilization during the preparation of the swelling witnesses.

The addition of TFV to these polymers leads to important changes in their PSD. The incorporation of TFV to chitosan produces an increase in porosity and the pore sizes become smaller. These changes continue if the concentration of TFV increases, due to the TFV’s structuring power over chitosan, which improves the system’s capacity to imbibe water and causes the appearance of pores when water is removed during the lyophilization process for obtaining the swelling witnesses. Data obtained from the swelling test indicate that C30 and C100 generated systems with higher swelling, and the number of pores is related to this value.

For the locust bean gum polymer, the addition of TFV causes little change in both pore volume and PSD, although a slight increase in pore size is observed at higher concentrations of TFV in the polymer. In this case it can be concluded that the presence of the drug does not modify the behaviour of this polymer in the medium. In other words, locust bean gum gel is so robust that its structure—and therefore its porosity—is not conditioned by the presence of TFV. This can be related with the results of hardness evaluation, as it was shown that locust bean gum-based tablets were the least affected by the presence of drug. Finally, the incorporation of TFV to the pectin polymer produces a significant decrease in the total pore volume and a smaller pore size; these changes are also independent of the amount of TFV. One possible explanation is that even though the presence of TFV does not modify either the rate or the amount of medium imbibed by pectin in its gelling process (the swelling profiles for P, P30 and P100 are practically overlapping), the visual observation of the systems during the swelling test revealed that swelled systems with the drug (P30 and P100) were more compact than P swelled tablets.

The addition of TFV to the polymer mixtures (i.e., CL and CP) caused major changes in the PSD data. In the case of CL tablets, the incorporation of TFV (CL30 and CL100) leads to less porous swelled systems; in other words, lower pore volume and pore diameter. These data agree with the data from the swelling test, as CL30 and CL100 had a lower SR_max_ and slower uptake rates of the medium (higher *t*_max_), indicating that the presence of TFV modifies the arrangement of the chains in both polymers in the water uptake process and reduces their swelling capability compared to the CL batch. The addition of TFV to CP batches forms systems (CP30 and CP100) with smaller pores (lower pore diameter) but their pore volume data are higher than the corresponding CP system, which can be attributed to TFV’s structuring power over chitosan. The entry of the aqueous medium in the system is therefore hindered during the swelling process, and although the swelling capacity is not modified by the presence of TFV (similar *SR*_max_ data for CP, CP30 and CP100 batches), the time required to achieve *SR*_max_ is longer (higher *t*_max_ of CP30 and CP100 respect to CP data). This is the reason that smaller pores are observed in swelling witnesses of CP30 and CP100.

In summary, the addition of a gelling polymer to chitosan-based systems leads to a modification of the microstructure of the system due to the redistribution of the polymer chains during the swelling process caused by the interaction between the polymers.

Figure 6 shows the microstructure of all the swelling witnesses that corroborate the porosity results described above.

### 3.5. Drug Release

**Figure 7:**
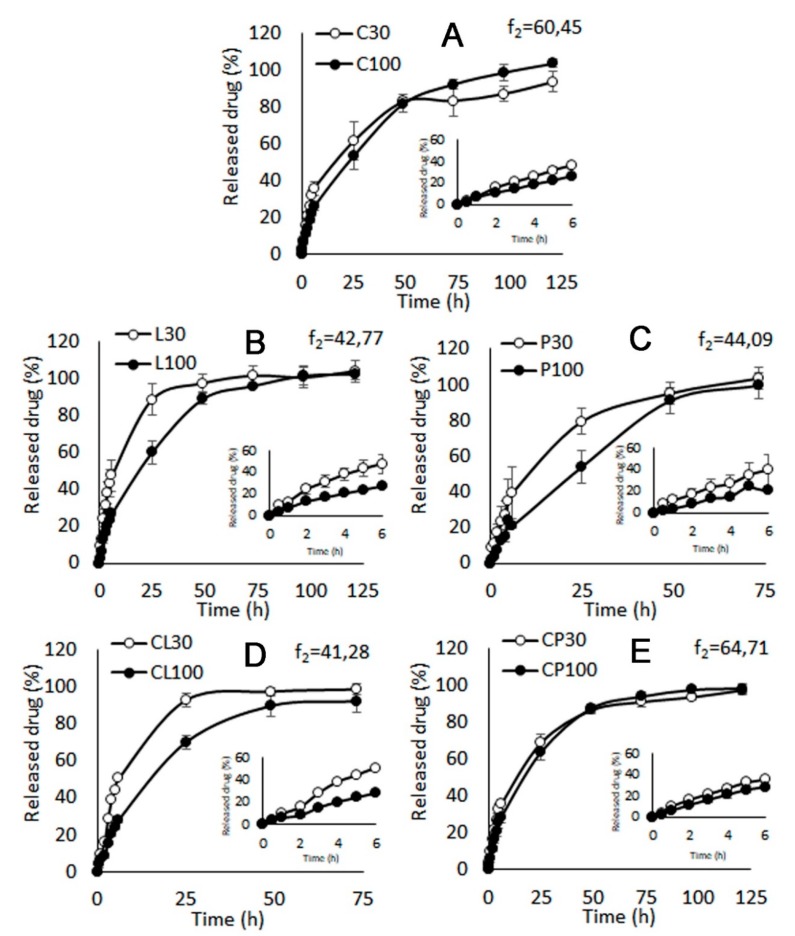
Drug release profiles in SVF from C (**A**), L (**B**), P (**C**), CL (**D**) and CP (**E**) batches containing 30 mg and 100 mg of TFV. Similarity factors are included in each graphics. The first six hours are shown in detail in the graphics in the bottom right corner.

The TFV release profiles from the tablets are shown in Figure 7. Sustained release of over 24 h are observed in all cases. However, the TFV release profiles showed to be conditioned by the composition of the tablets.

According to Figure 7A, chitosan-based systems (C30 and C100) are able to release the drug over 120 h, because although the system starts eroding from 24 h (as seen in the swelling profiles in Figure 3), the drug continues to be released from the fragments of the eroded tablets. There are no significant differences between C30 and C100 batches; this may be related to the porosity results, which indicate that the volume of pores is proportional to the amount of drug, so the release of TFV through the pores is independent of the amount of drug

The TFV dissolution profiles from the locust bean gum batches (L30 and L100) in Figure 7B show the sustained releases of TFV for up to 120 h due to a progressive diffusion of the drug through the high-consistency gel generated in the medium according to Chakravorty et al. [45]. Significant differences can be observed in the release profiles, which can be attributed to the drug being released by diffusion; there is more drug in the gel when the medium penetrates into the system, so more time is required to dissolve the drug. Since the volume of pores in the system is constant regardless of the amount of drug in the tablet, the greater the amount of TFV in the tablet, the slower the drug release through the gel.

Drug release from batches based on pectin (P30 and P100) lasts a maximum of 72 h, as seen in Figure 7C. The low consistency of the gel formed allows a greater entry of SVF, so the drug diffuses faster through it. In this case, there are also significant differences depending on the amount of drug. As the drug is also released through diffusion, higher concentrations of TFV within the tablet slow its dissolution in the small amount of fluid in the gel. According to the PSD results, there are no differences either in pore volume or pore size for P30 and P100, so the release of the drug through the pores is slower for larger amounts of drug.

Figure 7D shows a drug release of up to 72 h in the case of batches of chitosan combined with locust bean gum (CL30 and CL100). This is shorter than observed in the batches prepared with each of the polymers separately. The explanation is that CL30 and CL100 tablets are unable to gel and undergo the destructuring that is responsible for releasing the drug. This destructuring of CL30 and CL100, once they are introduced in SVF, can be seen in the micrographs of the corresponding swelling witnesses (Figure 6).

According to Figure 7E, batches prepared with the association of pectin and chitosan (CP30 and CP100) show overlapping TFV controlled-release profiles that are not dependent on the amount of drug they contain. This is because the combination of both polymers forms a polyelectrolyte complex, as previously described in the swelling test section, creating a robust gelled system able to control the release of TFV during 120 h. Of all the systems evaluated, those based on the combination of chitosan and pectin are the only ones with a sustained TFV release profile that does not depend on the amount of drug in the tablet. This mixture of polymers can therefore be said to form the most robust formulation in the evaluation.

### 3.6. Mucoadhesion Assessment

**Figure 8:**
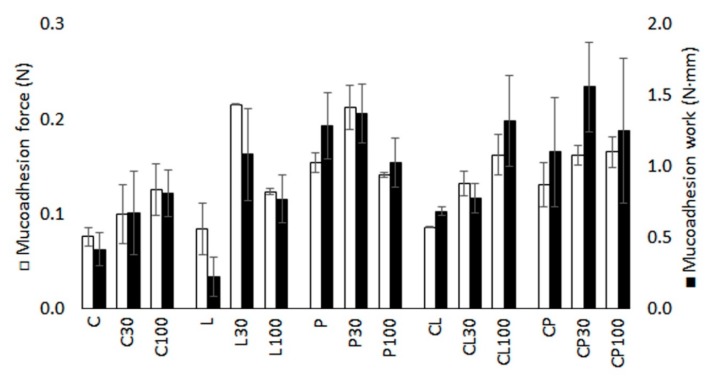
Mucoadhesion force and mucoadhesion work observed for the batches prepared on bovine vaginal mucosa.

Figure 8 contains the results of the mucoadhesion forces and mucoadhesion works for the batches. It can be seen that all the blank formulations (C, L, P, CL and CP) are able to attach to the vaginal mucosa, and force values of between 0.075 N and 0.16 N are required to detach the tablets. This confirms the mucoadhesiveness of all the polymers assayed, whose mechanisms of adhesion have been previously described (chitosan through electrostatic interactions [46] and pectin and locust bean gum through hydrogen bonds [58]). Pectin is the polymer with the highest mucoadhesion force and work values in quantitative terms compared to locust bean gum and chitosan. When chitosan and locust bean gum are mixed (CL tablets), the mucoadhesion force and work are similar to that obtained from tablets prepared with the unmixed polymers (C tablets and L tablets). However, the association of chitosan and pectin (CP tablets) has a mucoadhesion force and work close to those obtained from tablets prepared with pectin exclusively (P tablets), confirming the high mucoadhesiveness of pectin even when mixed. The addition of TFV to the systems leads in all cases to an increase in the mucoadhesion force and work. The mucoadhesion force cannot be related to the amount of drug in the systems. In fact, the results of the ANOVA analysis indicate that there are no significant differences in the mucoadhesion force and work among the blank batches (C, L, P, CL and CP), those loaded with 30 mg (C30, L30, P30, CL30 and CP30) and those containing 100 mg (C100, L100, P100, CL100 and CP100) of TFV, as shown in Table 4.

Once it was verified that all the formulations had mucoadhesive properties, the next step was to determine how long they remained bonded to the vaginal mucosa.

**Figure 9:**
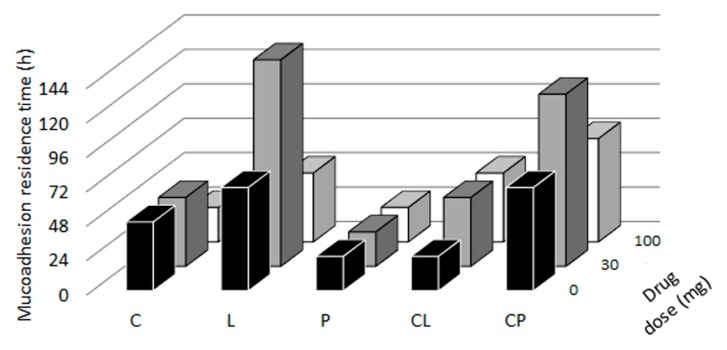
Mucoadhesion residence times for the batches on bovine vaginal mucosa.

Figure 9 shows the residence times of the batches. All the blank systems remain mucoadhered for at least 24 h. Chitosan-based tablets (C tablets) are lost through erosion due to the destructuration of the system. Tablets containing locust bean gum (L tablets) get detached from the mucosa while they are still embibing water, so the whole swollen tablet is lost. Tablets with pectin (P tablets) showed the lowest residence times, as it formed a fluid gel unable to maintain its structure, and adherence to the mucosa, so the detachment occurs when the *SR*_max_ determined in the swelling tests (Section 3.3) is reached. The combination of chitosan and locust bean gum leads to a lower residence times due to the destructuration of the system described in the swelling studies (Section 3.3), while the combination of chitosan and pectin (CP tablets) had the longest mucoadhesion residence times, as the tablets do not detach from the mucosa, so the system is dissolved in the medium still being attached to the surface of the mucosa. This confirms the robustness of the chitosan-pectin combination due to the formation of the previously described polyelectrolyte complex [37] and also to the swelling and porosity data. However, as in the case of the mucoadhesion force measurements, the ANOVA results indicate that there are no significant differences in mucoadhesion residence times among the blank batches (C, L, P, CL and CP), those loaded with 30 mg (C30, L30, P30, CL30 and CP30) and those containing 100 mg (C100, L100, P100, CL100 and CP100) of TFV, as shown in Table 5.

Although the TFV controlled-release profiles, mucoadhesion force and mucoadhesion residence times are the parameters used to select the best formulation, the patient’s comfort is a factor that cannot be overlooked when seeking to improve adherence to the treatment. The most suitable formulation is therefore the one with a moderate swelling behaviour that is able to remain in the vaginal mucosa while the drug is being released. Although chitosan-based batches swell very little, they disintegrate very quickly; this would hinder their presence at the site of action since the fragments would be expelled from the vagina, leading to reduced mucoadhesion times [29]. In the case of locust bean gum and pectin batches, although the structure is maintained during the SVF uptake process, the swelling is so great that they cannot strictly be considered an option for vaginal administration, since the patient’s discomfort would compromise their therapeutic compliance [19]. Although the combination of chitosan and locust bean gum reduces the maximum swelling, the formulation dissolves quickly in the medium, so vaginal leakage could be expected to be high. However, the association of chitosan and pectin generates systems with a moderate swelling but which maintain their structure for prolonged periods of time, so they may be good candidates to fulfil the objective of this work. Vaginal turnover must also be taken into account. This is the physiological mechanism whereby any possible harmful elements such as pathogens or foreign particles are expelled from the vaginal environment [59]. This mechanism represents a limitation for mucoadhesive vaginal systems, as this cyclical process occurs after 96 h and barely allows the permanence of formulations during prolonged periods in in vivo studies. Fortunately, CHP systems for the controlled release of TFV (C30 and C100) deliver almost the entire drug amount before vaginal turnover occurs, and the mucoadhesion time exceeds this period.

## 4. Conclusions

The association of pectin and chitosan generates an electrostatic interaction between both polymers in SVF, thus obtaining robust and highly-structured gelling systems in this medium, with a moderate swelling that ensures therapeutic compliance, and a mucoadhesion residence time and controlled release of tenofovir for 4 days, the time corresponding to vaginal turnover.

It has been demonstrated that the antiretroviral drug tenofovir can be integrated in the matrix of these tablets regardless of its quantity, so they can also be said to be robust systems that are not modified by the amount of drug they contain. The tablets based on chitosan and pectin are therefore interesting candidates for the prevention of sexual transmission of HIV through the controlled release of tenofovir for 4 days.

## Figures and Tables

**Table 1:** Composition of the batches prepared in mg/tablet.

Batch	Chitosan	Locust Bean Gum	Pectin	MgS	TFV
C	290			3	
C30	290			3	30
C100	290			3	100
L		290		3	
L30		290		3	30
L100		290		3	100
P			290	3	
P30			290	3	30
P100			290	3	100
CL	145	145		3	
CL30	145	145		3	30
CL100	145	145		3	100
CP	145		145	3	
CP30	145		145	3	30
CP100	145		145	3	100

**Table 2:** Results obtained from the characterization of the polymers employed.

	[*η*]	*K* (dL/g)	α	*M*_w_ (kDa)	DD (%)	DE (%)
Chitosan	24.74	9.30 × 10^−3^ [45]	0.76 [45]	3.21 × 10^1^	54.73 ± 4.26	
Locust bean gum	16.42	3.04 × 10^−4^ [43]	0.80 [43]	2.78 × 10^3^		
Pectin	20.00	1.4 × 10^−6^ [44]	1.34 [44]	2.19 × 10^2^		79.91 ± 1.66

**Table 3:** Data of thickness (mm), diameter (mm) and weight (mg) of the tablets prepared.

Batch	Thickness (mm)	Diameter (mm)	Average Weight (mg)	Theoretical Weight (mg)
C	1.683±0.006	13.027 ± 0.021	292.00 ± 0.82	293
C30	1.857 ± 0.040	13.033 ± 0.015	323.23 ± 0.47	323
C100	2.340 ± 0.052	13.070 ± 0.104	394.45 ± 0.07	393
L	1.833 ± 0.029	13.020 ± 0.104	293.17 ± 0.32	293
L30	1.970 ± 0.020	13.120 ± 0.010	322.40 ± 1.27	323
L100	2.367 ± 0.006	13.097 ± 0.012	392.57 ± 0.68	393
P	1.747 ± 0.006	13.057 ± 0.006	292.63 ± 1.10	293
P30	1.953 ± 0.029	13.090 ± 0.010	323.67 ± 1.07	323
P100	2.347 ± 0.042	13.057 ± 0.006	392.40 ± 1.55	393
CL	1.977 ± 0.015	13.183 ± 0.015	292.90 ± 0.36	293
CL30	2.113 ± 0.015	13.150 ± 0.036	321.63 ± 0.32	323
CL100	2.410 ± 0.010	13.080 ± 0.017	391.70 ± 0.62	393
CP	1.700 ± 0.069	13.083 ± 0.015	292.10 ± 1.39	293
CP30	1.907 ± 0.029	13.053 ± 0.012	323.27 ± 0.55	323
CP100	2.277 ± 0.031	13.043 ± 0.006	393.77 ± 0.21	393

**Table 4:** Results of the ANOVA processing of the mucoadhesion forces of the batches; the factors are the nature of the polymer and the drug content. *p*-values below α = 0.05 indicate no significant differences.

	Polymer Nature	Drug Content	Interaction
*Mucoadhesion force*	9.47×10^-5^	9.61×10^-6^	1.01×10^-3^
*Mucoadhesion work*	8.28×10^-4^	3.12×10^-2^	3.61×10^-1^

**Table 5:** Results of the ANOVA processing of the mucoadhesion times of the batches; the factors are the nature of the polymer and the drug content. *p*-values below α = 0.05 indicate no significant differences.

Polymer Nature	Drug Content	Interaction
4.1×10^-6^	1.58×10^-3^	1.75×10^-2^
